# Beating the Odds: Successful Emergent Surgery After Acute Aortic Dissection in Takayasu Arteritis

**DOI:** 10.7759/cureus.103053

**Published:** 2026-02-05

**Authors:** Ana Raquel Nunes, Sílvia Morgado, Miguel Roxo, Célia Duarte, Cristina Ramos

**Affiliations:** 1 Anesthesiology, Unidade Local de Saúde de São José, E.P.E., Lisbon, PRT; 2 Anesthesiology, Unidade Local de Saúde do Alentejo Central, E.P.E., Évora, PRT

**Keywords:** acute aortic syndrome, aortic arch repair, cardiac anesthesia, frozen elephant trunk, perioperative management, pregnancy, takayasu arteritis, tevar, vascular surgery anesthesia

## Abstract

Acute aortic syndromes (AAS) encompass life-threatening conditions that require rapid diagnosis and specialized intervention. Among these, large-vessel vasculitides, such as Takayasu arteritis (TA), pose unique surgical and anesthetic challenges due to active vascular inflammation, tissue fragility, and possible multiorgan involvement. We report the perioperative management of a 34-year-old female, who was 20 weeks pregnant, with TA who presented with rapidly progressive aneurysmal dilation and dissection of the thoracic aorta, complicated by multiorgan failure. She underwent emergent hybrid repair of the aortic arch using a frozen elephant trunk (FET) prosthesis, followed by second-stage thoracic endovascular aortic repair (TEVAR) for a descending aortic aneurysm six months later. The initial perioperative period was complicated by hemodynamic instability, cardiac arrest, ventilatory challenges, renal failure, and fetal loss. Intraoperative management included transesophageal echocardiographic assessment, hemostatic optimization, and prioritization of cerebral and end-organ protection using moderate hypothermia and antegrade cerebral perfusion. The postoperative period was further complicated by severe myopathy, bilateral vocal cord paralysis, and limb ischemia secondary to vasopressor use.

Despite the complexity of the surgical case and the subsequent complications, the patient achieved a favorable functional recovery and remains under follow-up with stable aortic imaging findings. This report highlights the multidisciplinary challenges of managing extensive aortic disease, particularly when surgery is required during an active inflammatory phase. Careful preoperative assessment, individualized surgical and anesthetic strategies, advanced intraoperative monitoring, and prolonged postoperative support are essential to maximize outcomes in this high-risk population.

## Introduction

Acute aortic syndromes (AAS) comprise a spectrum of life-threatening conditions affecting the aortic wall with abrupt clinical onset. They include classic aortic dissection, intramural hematoma, penetrating atherosclerotic ulcer, and impending aneurysm rupture, with aortic dissection accounting for more than 90-95% of presentations [1]. The Stanford classification divides dissections into type A, involving the ascending aorta and requiring urgent surgery, and type B, confined to the descending thoracic aorta and usually managed medically or with thoracic endovascular aortic repair (TEVAR). Complementary systems, such as the DeBakey classification, further describe the anatomical extent and guide therapeutic strategy. Among AAS, those involving the ascending aorta and arch are associated with the highest mortality and require prompt intervention by experienced multidisciplinary teams [1-4].

Takayasu arteritis (TA) is a chronic, idiopathic granulomatous vasculitis that predominantly affects the aorta and its major branches, with a strong female predominance among individuals under 40 years of age [1,2]. Progressive inflammation may lead to stenosis, occlusion, aneurysm formation, and, in rare but catastrophic cases, dissection. Active inflammatory disease, identified by elevated erythrocyte sedimentation rate (ESR) and C-reactive protein (CRP) levels, in combination with imaging evidence of mural enhancement or wall thickening, is characterized by vascular fragility, rapid aneurysmal degeneration, and poor postoperative healing [3-5]. Although elective repair is ideally performed during quiescent phases confirmed through biomarkers and imaging, urgent surgery is often unavoidable when there is rapid aneurysm expansion, impending rupture, or malperfusion threatening organ function [4,6].

Pregnancy constitutes a significant modifier of aortic disease risk, introducing several physiological and hormonal changes that may trigger or accelerate aortic pathology. Physiological changes, such as increased blood volume and cardiac output, decreased systemic vascular resistance, hormonally mediated weakening of collagen and elastin, and progressive increases in aortic wall stress, may accelerate aneurysm growth or precipitate dissection, particularly in women with underlying vasculopathies such as TA. The coexistence of active inflammation and the hemodynamic load associated with pregnancy significantly heightens the risk of catastrophic aortic events [2].

Major surgery on the ascending aorta or arch in the context of vasculitis presents significant anesthetic challenges. Blood pressure monitoring must be individualized, as stenotic or occluded vessels may render certain limbs unreliable for accurate measurements; dual-site invasive monitoring (radial and femoral) is often required [7-9]. Advanced neuromonitoring, including processed electroencephalography [10], near-infrared spectroscopy (NIRS) [11], and, when available, evoked potentials, is crucial for guiding cerebral protection during cardiopulmonary bypass (CPB), selective antegrade cerebral perfusion, and periods of cardiocirculatory arrest. Moderate or deep hypothermia is used to reduce cerebral metabolic rate, whereas controlled rewarming is essential to prevent cerebral hyperemia and embolic complications. In addition, intraoperative management requires careful heparinization, point-of-care coagulation assessment (ROTEM/TEG) [12], systematic transesophageal echocardiography (TEE), and meticulous management of hemostasis before separation from CPB [5].

For aneurysms and dissections involving the proximal aorta, including the ascending aorta and aortic arch, surgical strategies have advanced significantly. The frozen elephant trunk (FET) technique, which combines open arch replacement with the deployment of a stent graft into the descending aorta, has become particularly valuable in extensive or complex disease, providing durable proximal repair and creating optimal conditions for subsequent endovascular interventions. In patients with TA, where lesions often extend across multiple aortic segments, staged hybrid strategies are increasingly favored [7-9]. This report describes the perioperative management of a young pregnant woman with active TA and rapidly progressive aneurysmal degeneration of the thoracic aorta, characterized by short-interval aneurysm expansion and structural instability, who underwent emergency arch repair with a FET prosthesis, followed by a staged TEVAR. The report highlights the interplay between systemic inflammation, pregnancy-related physiology, and the complex technical and anesthetic considerations required in high-risk aortic surgery.

Informed consent was obtained from the patient for the publication of this case report and accompanying images.

## Case presentation

Patient information and history of present illness

We present the case of a 34-year-old woman from a sub-Saharan African country, living in Portugal for a year, with no known personal or family history of cardiovascular or autoimmune disease, and who denied tobacco, alcohol, or illicit drug use. Her medical history was notable for long-standing irregular menstrual cycles.

After arriving in Portugal, the patient presented to the emergency department with worsening chest pain. On examination, she was febrile and tachycardic, emaciated but adequately hydrated, with normal cardiopulmonary auscultation, no carotid bruits, no peripheral edema, and no objective signs of inflammatory arthritis. Laboratory testing revealed a markedly elevated CRP level, the absence of leukocytosis, and microcytic hypochromic anemia. CT imaging demonstrated aortic dissection, prompting transfer to the cardiothoracic surgery service. She reported being in her usual state of health until approximately 8-12 months earlier, when she had developed intermittent, non-exertional chest pain radiating to the interscapular region that had progressively worsened and begun to occur at rest, associated with anorexia, unintentional weight loss, night sweats, subjective fever, and inflammatory-type arthralgia of the hands and feet. Approximately six months before presentation, while still in her country of origin, she had experienced an episode of severe chest pain preceded by fever.

**Figure 1:**
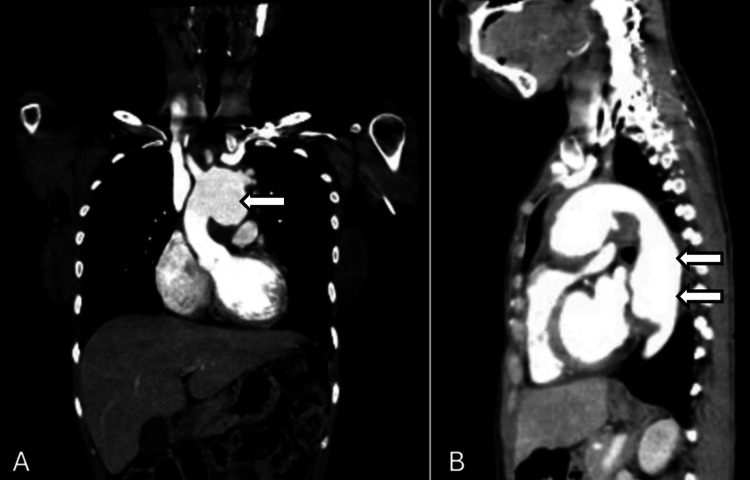
Initial CT angiography showing dilation of the aortic arch, descending thoracic aortic aneurysm and involvement of bilateral carotid and subclavian arteries A: coronal plane. B: Sagittal plane CT: computed tomography

An etiological workup excluded infectious causes. Immunological testing revealed positive antinuclear antibodies with a cytoplasmic pattern, while other autoimmune markers were negative. Initial imaging evaluation using CT angiography (Figure 1) revealed marked dilation of the aortic arch (maximum 4.7 cm) with multiple saccular aneurysms (largest 2.5 cm), a proximal descending thoracic aortic aneurysm measuring 4.1 cm with a mural thrombus, affecting the left carotid and subclavian arteries. Transthoracic echocardiography demonstrated a pseudoaneurysm between the aorta and pulmonary artery (45 × 37 mm), a possible dissection flap in the descending thoracic aorta, left ventricular ejection fraction of 47% with mild global hypokinesia, and a small pericardial effusion. PET-CT highlighted significant vascular inflammation affecting the thoracic aorta, left common carotid, and left subclavian arteries.

Based on clinical, laboratory, and imaging findings, a diagnosis of probable Takayasu arteritis was established (The European Alliance of Associations for Rheumatology (EULAR) classification score: 6) [13]. Treatment was initiated with oral prednisolone at 1 mg/kg/day (60 mg), continued for approximately five months, including tapering. She was discharged with outpatient internal medicine follow-up, and methotrexate 7.5 mg orally once weekly was initiated. Concomitant medications included omeprazole 10 mg orally once daily and carvedilol 25 mg orally once daily.

Presentation and ICU course

Six months after the initial evaluation, the patient presented for a scheduled CT, which she could not tolerate due to orthopnea. Additionally, she reported feeling feverish, progressive fatigue, and severe chest pain. She was admitted for inpatient care. The initial physical examination revealed tachypnea, absent heart sounds, distended jugular veins, hypoxemia (SpO₂: 85% on ambient air), tachycardia (HR: 140 bpm), and a blood pressure of 132/98 mmHg. Given progressive worsening, the patient was admitted to the ICU.

Shortly after admission, the patient progressed to multiorgan failure. Respiratory involvement included severe hypoxemic respiratory failure (PaO₂/FiO₂ ≈70), requiring endotracheal intubation and mechanical ventilation. Concurrently, she exhibited hemodynamic instability and hyperlactatemia, necessitating variable but increasingly higher doses of noradrenaline support (approximately 0.94 mcg/kg/min). The clinical course was further complicated by acute kidney injury, which required continuous venovenous hemodiafiltration, and congestive hepatopathy, evidenced by elevated liver enzymes and mild coagulopathy.

**Figure 2:**
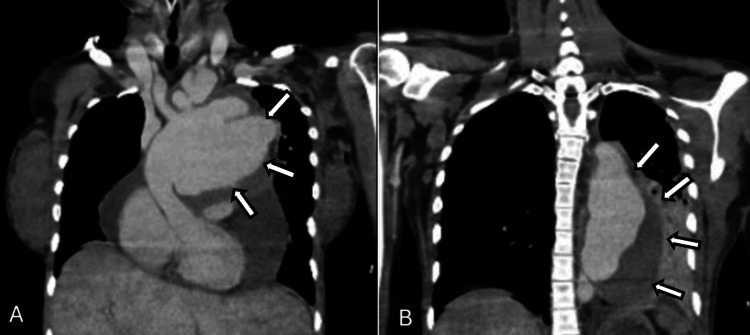
Preoperative CT angiography showing extensive pseudoaneurysm of the aortic arch (A) and descending aorta (B) CT: computed tomography

After initial stabilization, CT angiography (Figure 2) showed rapid aneurysmal progression compared to previous studies: ascending aorta 13.4 cm, aortic arch 9.1 cm, descending aorta 8.2 cm, along with a large pericardial effusion (53 mm). Additional findings included hepatic congestion, mild ascites, and an incidental 20-week intrauterine pregnancy.

Supportive therapy included broad-spectrum antibiotics (piperacillin-tazobactam adjusted to the renal function), intravenous hydrocortisone (200 mg/day), and strict organ support measures (namely dialysis). Given the imminent risk of aortic rupture, the multidisciplinary team (cardiac surgery, vascular surgery, anesthesia, and rheumatology) recommended urgent aortic repair, despite prohibitive operative risk.

Preoperative cardiac arrest

On the following day, while being prepared for transfer to the operating room, the patient experienced cardiac arrest in asystole/pulseless electrical activity (PEA). Advanced life support was initiated, and return of spontaneous circulation was achieved after approximately eight minutes. Post-arrest brain CT showed no major hypoxic-ischemic injury.

First operation - frozen elephant trunk procedure 

The patient underwent a total arch replacement on the day after using a ThoraflexTM Hybrid graft [14], including reimplantation of the brachiocephalic trunk and left common carotid artery, with conduit construction for the left subclavian artery.

Anesthesia and Monitoring

General balanced anesthesia was maintained using a combination of propofol, fentanyl, rocuronium, and midazolam. A multimodal monitoring strategy was implemented, which included bispectral index (BIS) [10], NIRS [11], and continuous TEE. Hemodynamic assessment was performed using both invasive radial and femoral arterial pressure monitoring, along with central venous pressure measurement. Core temperature was monitored at both bladder and esophageal sites to ensure thermal stability throughout the procedure.

Cardiopulmonary Bypass (CPB)

The total CPB duration was 247 minutes (four hours and seven minutes). Deep hypothermic circulatory arrest was maintained for 60 minutes at a core temperature of 22 °C, during which selective antegrade cerebral perfusion was provided via the left common carotid artery to ensure neurological protection.

Intraoperative Complications

The intraoperative period was complicated by a cardiac arrest (asystole) during patient positioning, which required six minutes of cardiopulmonary resuscitation before achieving the return of spontaneous circulation. Significant blood loss occurred, which was managed through a goal-directed transfusion protocol and cell-saver reinfusion. The patient received three units of packed red blood cells, four units of fresh frozen plasma, and two pools of platelets. Hemostasis was further optimized with the administration of 4 g of fibrinogen and 1 g of tranexamic acid, strictly guided by point-of-care rotational thromboelastometry (ROTEM) [12].

Postoperative Course 

The postoperative course was complicated and required prolonged intensive care. The patient remained under mechanical ventilation for 16 days and required extended vasopressor (maximum 0.8 mcg/kg/min) and inotropic support (maximum 1.4 mcg/kg/min), which was weaned off six days after surgery. Renal function deteriorated, requiring renal replacement therapy with dialysis for two days. Following extubation, bilateral vocal cord paresis was diagnosed, likely secondary to recurrent laryngeal nerve injury. Furthermore, the patient developed lower limb ischemia, attributed to the prolonged use of high-dose vasopressors. Regarding the obstetric outcome, an evaluation on the day after the surgery confirmed intrauterine fetal demise; spontaneous abortion occurred later that day without further hemorrhagic complications.

Recovery and Rehabilitation

The patient’s clinical status improved gradually, allowing for successful weaning from mechanical ventilation and the initiation of a multidisciplinary rehabilitation program. She was transferred to the cardiac surgery ward on the 13th postoperative day. However, the previously noted lower limb ischemia progressed to distal necrosis of the right toes; consequently, 48 days after the initial surgery, she underwent a distal phalanx amputation of the second toe under regional anesthesia (sciatic nerve block). To address persistent dysphonia and dysphagia secondary to vocal cord paresis, speech therapy was instituted with favorable results. Functional recovery progressed steadily, and at the time of discharge, the patient demonstrated independent ambulation, full cognitive recovery, and overall clinical stability. A summary of laboratory assessment throughout the perioperative period is presented in Table 1.

**Table 1:** Longitudinal laboratory assessment throughout the perioperative period Infectious serologies included cytomegalovirus, Epstein-Barr virus, *Toxoplasma gondii, Chlamydia species, Mycoplasma, Borrelia, Coxiella*, and *Yersinia*; syphilis and *Brucella*. The autoimmune panel included antinuclear antibodies, extractable nuclear antigens, antineutrophil cytoplasmic antibodies (proteinase 3/myeloperoxidase), antiphospholipid antibodies, and rheumatoid factor ICU: intensive care unit; FET: frozen elephant trunk; ESR: erythrocyte sedimentation rate; CRP: C-reactive protein; PT: prothrombin time; INR: international normalized ratio; aPTT: activated partial thromboplastin time; AST: aspartate aminotransferase; ALT: alanine aminotransferase; LDH: lactate dehydrogenase; CK: creatine kinase; ANA: antinuclear antibodies

Parameter	Diagnosis	ICU admission	Pre-FET	D1 post-FET	D2 post-FET	Discharge	Units	Reference range
Hemoglobin	7.7	9.7	9.8	10.7	10.2	11.1	g/dL	12.0–15.0
Leukocytes	8.06	7.86	11.14	12.57	16.59	8.99	×10⁹/L	4.5–11.0
Neutrophils	63.7	71.5	85.7	88.9	90.5	60.7	%	40–75
Platelets	457	154	47	77	39	>104	×10⁹/L	150–450
ESR	91	37	—	—	—	—	mm/h	<16
CRP	133.1	65.4	—	—	109	1.7	mg/L	<5.0
PT	16.7	—	25.4	18.2	—	11.5	s	9.4–12.5
INR	1.45	—	2.23	1.59	—	0.96	—	0.8–1.2
aPTT	32.8	—	34.4	30.4	—	16.6	s	25.1–36.5
Serum urea	22	14	27	48	84	34	mg/dL	16.6–48.5
Serum creatinine	0.60	0.47	1.00	1.68	1.96	0.54	mg/dL	0.51–0.95
Serum sodium	—	135	140	149	149	141	mEq/L	136–145
Serum potassium	—	3.7	3.7	4.2	4.3	3.8	mEq/L	3.5–5.1
Total bilirubin	0.21	0.40	—	—	4.8	—	mg/dL	<0.90
AST	—	11	3250	—	427	—	U/L	<32
ALT	—	7	1112	—	223	—	U/L	<33
LDH	—	359	3969	—	1226	—	U/L	135–214
CK	19	26	—	1484	1176	—	U/L	<170
High-sensitivity troponin T	—	9.0	—	506	557	—	ng/L	<14
Autoimmune panel	ANA positive	—	—	—	—	—	—	—
Extensive infectious screening	Negative	—	—	—	—	—	—	—

Second operation - TEVAR 

Six-month follow-up CT revealed rapid growth of a descending aortic aneurysm (≈10 cm). She underwent elective TEVAR under general anesthesia. The surgical technique included percutaneous left femoral and right humeral access, with retroperitoneal exposure of the right iliac artery for conduit construction.

Anesthesia and Monitoring

General anesthesia was induced with a combination of propofol, fentanyl, and rocuronium. Intraoperative additional monitoring included invasive arterial pressure, bispectral index, core temperature, activated clotting time (ACT), and serial arterial blood gas analysis. Hemodynamic stability was prioritized, maintaining a mean arterial pressure above 80 mmHg through a titrated noradrenaline infusion. Intraoperative blood loss was moderate, necessitating the transfusion of one unit of packed red blood cells. For postoperative transition and sedation, a dexmedetomidine infusion was initiated

Outcome

**Figure 3:**
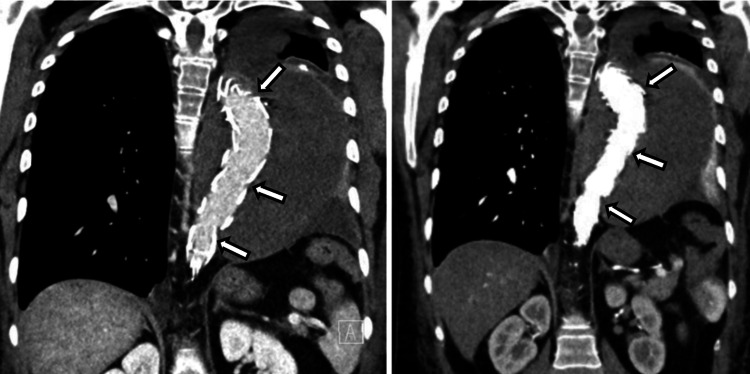
Post-TEVAR CT angiography showing sealed stent graft and no endoleak TEVAR: thoracic endovascular aortic repair; CT: computed tomography

The patient’s total postoperative hospital stay lasted 10 days, which included six days in the ICU. At the time of discharge, she exhibited no neurological deficits. Follow-up CT angiography confirmed a well-positioned and sealed stent graft, with no evidence of endoleaks (Figure 3).

Discharge to Rehabilitation

The patient was hemodynamically stable, ambulating independently, and free of heart failure symptoms.

## Discussion

Takayasu arteritis is a chronic large-vessel vasculitis with significant anesthetic and surgical implications due to progressive inflammation and involvement of the aorta and its major branches. Although corticosteroids and immunosuppressive agents remain the cornerstone of therapy, approximately one-third of patients ultimately require revascularization or aneurysm repair because of progressive stenosis, dilation, or dissection [15-17].

Surgical approach

Acute aortic syndromes - particularly type A aortic dissection - represent surgical emergencies due to their high lethality and the risk of catastrophic complications, including rupture, tamponade, and end-organ malperfusion. The American Heart Association (AHA) recommends immediate surgical repair, as isolated medical management is associated with profoundly increased mortality. Similarly, the European Association for Cardio-Thoracic Surgery (EACTS) and the Society of Thoracic Surgeons (STS) emphasize that each hour of delay increases mortality by approximately 0.5% [18,19]. 

Management becomes more complex in the setting of large-vessel vasculitis such as TA. Active inflammation markedly increases surgical risk due to friable tissues, impaired wound healing, and hemodynamic instability. Current EACTS/STS guidelines note that open surgical repair provides superior long-term outcomes in TA; however, the risk of graft failure or anastomotic complications increases when surgery is performed during active disease, underscoring the importance of preoperative disease control when feasible [15]. 

Hybrid Repair and the Role of the Frozen Elephant Trunk (FET)

For extensive disease involving the aortic arch or descending thoracic aorta, hybrid approaches, particularly the FET procedure, are indicated, especially when the primary tear arises in the distal arch or proximal descending aorta [14,19,20]. The FET technique facilitates simultaneous arch replacement and distal aortic stent grafting, improving false-lumen thrombosis and long-term aortic remodeling. However, neurological complications, including spinal cord ischemia, remain a recognized risk, especially with longer stent grafts (>15 cm) [14,20]. 

Endovascular Therapy and TEVAR in Takayasu Arteritis

TEVAR has emerged as an important alternative in selected patients with descending aortic involvement. While TEVAR offers reduced operative morbidity and avoids deep hypothermic circulatory arrest, its use in TA remains controversial due to high rates of restenosis and stent failure caused by inflammatory-mediated arterial remodeling; risk of stent migration or endoleak in patients with non-calcified, inflamed aortic tissue; and reduced long-term durability compared with open repair, particularly during active vasculitis [5].

Nevertheless, TEVAR can be life-saving when the patient is hemodynamically unstable; when open repair carries a prohibitive surgical risk, when the pathology is confined to the descending thoracic aorta, or when bridging therapy is required until inflammation is controlled. Several case series suggest acceptable short-term outcomes with TEVAR in TA, although reintervention rates remain higher than with open surgery. Because the patient in this case required urgent arch and ascending repair, TEVAR alone was not feasible, and the FET strategy provided the most comprehensive and durable solution.

Timing of Surgery

Elective repair is preferred when inflammatory markers and imaging (MRI, PET/CT) indicate disease quiescence. Emergency surgery during active inflammation, as required in this case, carries higher mortality and postoperative complications but may be unavoidable in scenarios of rapid aneurysmal expansion or hemodynamic compromise [16,21]. 

Takayasu arteritis and pregnancy

Our patient exhibited significant disease exacerbation during pregnancy, culminating in acute aortic dissection. Pregnancy induces marked hormonal and hemodynamic changes that increase susceptibility to vascular complications, particularly in women with pre-existing aortopathies such as TA [15,21,22]. The ACC/AHA guidelines recognize pregnancy as a high-risk period for aortic dissection in patients with underlying aortic disease, with most dissections occurring during the third trimester or early postpartum period [22]. The STS highlights that rapidly enlarging aortic diameters during pregnancy should prompt multidisciplinary assessment and consideration of prophylactic surgical intervention in selected cases [19]. 

Active TA during pregnancy is strongly associated with adverse maternal and fetal outcomes, including hypertension, preeclampsia, miscarriage, and fetal growth restriction. Disease activity at conception and during gestation is the most significant predictor of poor outcomes, necessitating close monitoring, blood pressure control, and coordinated care among obstetrics, cardiology, and rheumatology teams. Pregnancies occurring after the diagnosis of TA or during active disease carry significantly greater risk than pregnancies occurring before diagnosis, primarily due to uncontrolled inflammation and hemodynamic instability [15,23]. The acute dissection in this case underscores the need for rigorous surveillance and guideline-directed management in pregnant patients with known vasculitis [19,22,23].

Anesthetic approach

Anesthetic management for FET repair in TA requires careful consideration of altered vascular anatomy, arterial stenoses, and increased risk of neurologic injury. Significant inter-limb blood pressure differences are common and necessitate the placement of dual invasive arterial lines, typically radial and femoral, to ensure reliable hemodynamic monitoring. Ultrasound guidance is essential to minimize complications in stenotic or fragile vessels [15,24]. 

Hemodynamic management requires a delicate balance, as abrupt blood pressure changes can precipitate dissection propagation or organ malperfusion, while the use of vasoactive drugs during critical periods highlights the crucial trade-off between maintaining mean arterial pressure and the significant risk of peripheral ischemia in already compromised vessels. Cerebral and organ perfusion must be maintained throughout cardiopulmonary bypass and hypothermic circulatory arrest. NIRS and TEE provide valuable real-time assessment [24,25].

Neuromonitoring strategies (including NIRS, processed electroencephalography, and somatosensory or motor evoked potentials) help reduce neurological complications, which are comparatively high in FET procedures [25]. Neuroprotective measures include moderate-to-deep hypothermia (20-28 °C), selective antegrade cerebral perfusion, and maintenance of adequate mean arterial pressure during circulatory arrest [20,21]. 

Patients with autoimmune disease exhibit increased thromboembolic risk. Standard anticoagulation includes unfractionated heparin (activated clotting time >400 seconds). The American College of Rheumatology conditionally recommends low-dose aspirin for cerebrovascular involvement, though perioperative use must be individualized [16]. Coagulation monitoring using viscoelastic testing is particularly valuable in the context of active vasculitis [25]. 

Immunosuppression considerations

Immunosuppressive optimization remains a cornerstone of perioperative management in Takayasu arteritis, particularly when major aortic intervention is required during active disease. Corticosteroids represent the first approach during exacerbations, often complemented by steroid-sparing agents such as methotrexate, azathioprine, or biologics targeting IL-6 to achieve disease quiescence afterwards. Adequate inflammatory control is associated with reduced risks of anastomotic complications, restenosis, and early structural graft failure, although immunosuppression must be balanced against increased infection risk and impaired wound healing [16,17,26].

Potential complications

Major aortic repair using the FET technique or TEVAR carries substantial perioperative risk, which is amplified in patients with large-vessel vasculitis. Neurological injury, particularly stroke or spinal cord ischemia, remains one of the most significant complications, especially with extensive arch replacement or long endovascular stent grafts. Hemodynamic instability, challenging arterial access, and difficulties interpreting blood pressure gradients across stenotic or inflamed vessels are well-described anesthetic concerns. Surgical complications include bleeding, anastomotic pseudoaneurysm, and stent-related issues such as endoleak or migration, which are more frequent in inflamed, non-calcified aortic segments [27].

In our case, the patient's course was marked by multiple complications, including cardiac arrests (pre- and intraoperative) successfully managed with prompt resuscitation; renal and hepatic dysfunction, requiring continuous dialysis and supportive management; bilateral vocal cord paralysis, a recognized risk of arch surgery due to recurrent laryngeal nerve traction, ischemia, or prolonged postoperative tracheal intubation; peripheral ischemia leading to toe amputation, related to high-dose vasopressors during obstructive shock, which emphasizes the delicate balance between maintaining perfusion and avoiding excessive vasoconstriction; and obstetric loss, likely multifactorial (hemodynamic instability and systemic inflammation).

These risks highlight the need for meticulous perioperative planning, advanced neuromonitoring, and rigorous postoperative surveillance. Multidisciplinary coordination among anesthesiology, cardiovascular surgery, autoimmune disease specialists, maternal-fetal medicine, and critical care teams is essential to optimize outcomes in this high-risk population.

## Conclusions

Aortic repair in TA presents distinctive challenges arising from active vascular inflammation, tissue fragility, and systemic involvement. Although emergency intervention during active disease should be reserved for life-threatening situations, this report demonstrates that successful outcomes are achievable through meticulous perioperative planning, advanced monitoring, and close multidisciplinary coordination. The combination of TEE, cerebral oximetry, and multimodal arterial pressure monitoring proved to be essential in tailoring perfusion and anesthetic strategies during CPB. Long-term management remains equally critical, requiring postoperative imaging surveillance and optimization of immunosuppressive therapy given the high risk of recurrent aneurysmal degeneration and stent-related complications. Rehabilitation and structured follow-up must also address neurological, functional, and vascular sequelae. This report highlights that even in the context of pregnancy, severe systemic dysfunction, and the need for two major aortic interventions over a short interval, coordinated multidisciplinary care can promote survival and recovery.
